# Simulation and Characterization of Nanoplastic Dissolution under Different Food Consumption Scenarios

**DOI:** 10.3390/toxics11070550

**Published:** 2023-06-23

**Authors:** Ying Wang, Zhongtang Wang, Xin Lu, Hongyan Zhang, Zhenzhen Jia

**Affiliations:** 1 College of Food Science and Engineering, Shandong Agricultural University, Tai’an 271018, China; 2 Shandong Provincial Key Laboratory of Animal Resistance Biology, Key Laboratory of Food Nutrition and Safety of Shandong Normal University, College of Life Sciences, Shandong Normal University, Jinan 250014, China

**Keywords:** food packaging, nanoplastic, dissolution, food consumption scenarios, fractional filtration

## Abstract

Understanding of the potential leaching of plastic particles, particularly nanoplastics (NPs), from food packaging is crucial in assessing the safety of the packaging materials. Therefore, the objective of this study was to investigate potential exposure risks by simulating the release of NPs from various plastic packaging materials, including polypropylene (PP), general casting polypropylene (GCPP) or metalized casting polypropylene (MCPP), polyethylene (PE), polyethylene terephthalate (PET), and polyphenylene sulfone (PPSU), under corresponding food consumption scenarios. Surface-enhanced Raman scattering (SERS) and scanning electron microscopy (SEM) were utilized to identify and characterize the NPs leached from plastic packaging. The presence of separated NPs was observed in PP groups subjected to 100 °C hot water, GCPP plastic sterilized at a high temperature (121 °C), and PE plastic soaked in 100 °C hot water, exhibited a distorted morphology and susceptibility to aggregation. The findings suggest that the frequent consumption of takeaway food, hot beverages served in disposable paper cups, and foods packaged with GCPP materials may elevate the risk of ingestion of NPs. This reminds us that food packaging can serve as an important avenue for human exposure to NPs, and the results can offer valuable insights for food safety management and the development of food packaging materials.

## 1. Introduction

Plastic has become the main material for food packaging owing to its functional properties, convenience, and cost-effectiveness [1]. However, environmental factors such as ultraviolet light exposure and mechanical wear can result in the degradation of plastic polymers into microplastics (MPs, particle size from 1 μm to 5 mm) or nanoplastics (NPs, particle size smaller than 1 μm) [2,3,4]. Due to the relatively mature and simple detection techniques and methods for MPs, there has been ample research conducted on them. Meanwhile, the accurate identification or detection of NPs poses greater challenges due to the size limitation and the ambiguous characteristics of plastic and non-plastic debris [5]. As a result, there is a paucity of studies on NPs, with a majority that focus on environmental samples and encompass the transportation, distribution, and toxicity of NPs in aquatic, terrestrial, and atmospheric environments [6,7]. Moreover, there is a significant dearth of studies on the pathways and hazards associated with human exposure to NPs [8]. The major route for NPs to produce toxic effects in the human body is the ingestion of food and drinking water. Recent studies have demonstrated the presence of NPs in food products packaged with plastic containers (such as bottled water, tea bags, and honey) [9,10,11,12], implying that NPs can be dissolved into food through plastic packaging and then ingested by humans.

The small size of NPs endows them with a greater potential to penetrate organisms and facilitates their accumulation in organs and tissues, thus bring higher health risks [6]. Therefore, it is urgent to investigate the consumption risks associated with NPs and advance the study of risk assessment. There is limited research on the ingestion of NPs by simulating a realistic diet. Hernandez et al. immersed food-grade plastic tea bags in hot water at 95 °C for 5 min and subsequently examined MPs and NPs in a leaching solution using scanning electron microscope analysis [9]. Fadare et al. introduced ultrapure water into a food-grade disposable plastic cup, agitated it for 2–3 min, and identified cubic, spherical, rod-shaped, and irregularly shaped NPs in the solution [13]. Other studies have suggested that MPs could be released from polypropylene (PP) infant feeding bottles when standard formula milk powder is dissolved in them [14]. The quantity of MPs released varies depending on factors such as the water temperature. In addition, Ranjan et al. confirmed that MPs can be dissolved from disposable paper cups after being filled with hot water for 15 min due to a hydrophobic polyphenylene film on the interior of the cups [15]. The above studies mostly emphasize the dissolution of MPs in food scenarios or analyze the dissolution of NPs or MPs by simulating only a single food scenario, which is far from real-world food scenarios [16].

**Figure 1:**
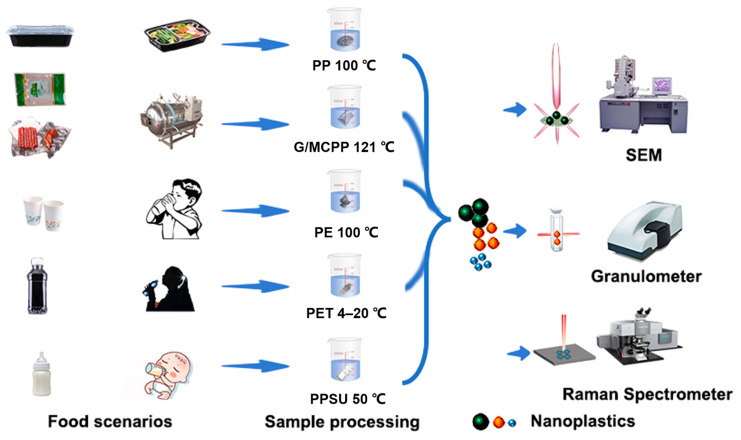
Schematic view of the dissolution of NPs in different food consumption scenarios.

In this study, more representative food consumption scenarios were simulated to investigate the dissolution behavior of NPs. Therefore, plastic packaging that comes into direct contact with food, such as PP takeaway boxes, general casting polypropylene (GCPP) or metalized casting polypropylene (MCPP) bags, polyethylene (PE) disposable paper cups, polyethylene terephthalate (PET) beverage bottles, and polyphenylene sulfone (PPSU) baby bottles, were utilized to simulate the processes of delivering take-out food, sterilization at high temperatures, the consumption of hot water or beverages, and the dissolution of milk powder, respectively. NPs were obtained via acid–base treatment, aqueous extraction, and fractional filtration, and the particles were characterized by scanning electron microscope (SEM), surface-enhanced Raman scattering (SERS), and particle size analyzer. A flow diagram of the research is illustrated in Figure 1.

## 2. Materials and Methods

### 2.1. Materials and Reagents

PP takeaway boxes, GCPP/MCPP packaging bags, PE disposable paper cups, PET beverage bottles, and PPSU baby bottles were purchased from RT-MART (Taipei, China). The aforementioned samples were not employed prior to the experiment. Polycarbonate filter membrane was sourced from Whatman (Little Chalfont, UK). Sodium hydroxide (NaOH), hydrochloric acid (HCl), magnesium sulfate (MgSO_4_), silver nitrate (AgNO_3_), and hydroxylamine hydrochloride (HONH_3_Cl) were purchased from Sinopharm Chemical Reagent Co., Ltd. (Shanghai, China).

### 2.2. Scenario Setup

**Figure 2:**
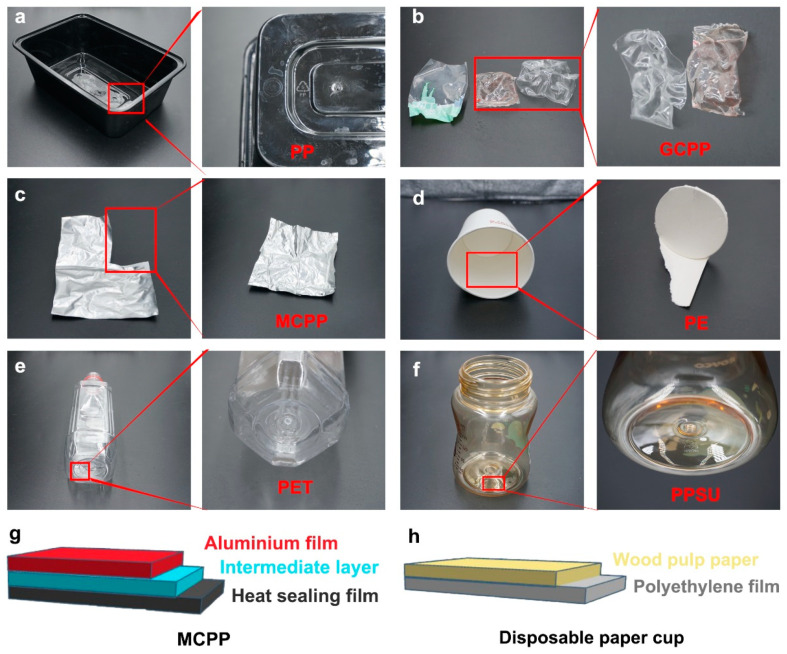
Profile display of the experimental model. (**a**) A PP takeaway box. Heat-sealing materials for food composite packaging with a composition of (**b**) GCPP and (**c**) MCPP. (**d**) A PE disposable paper cup. (**e**) A PET beverage bottle. (**f**) A PPSU baby bottle. Schematic illustration of MCPP (**g**) and disposable paper cup (**h**) structures.

PP material is often used for producing the packaging of takeaway food, so a PP takeaway box (Figure 2a) was selected and soaked in 500 mL ultrapure water at 100 °C (simulating take-out meals) for 20 min. A GCPP/MCPP packaging bag (Figure 2b,c,g) was soaked in 500 mL ultrapure water in a high-temperature sterilization pot of 121 °C (simulating the process of high-temperature sterilization) for 20 min. The main difference between GCPP and MCPP materials is that the latter is made with a polypropylene raw material for aluminum plating, whereas GCPP does not contain aluminum film. Both of these are often used as a heat-sealing material for food composite packaging. Pre-packaged food is usually subjected to a high-temperature sterilization process at 121 °C. The 500 mL of 100 °C ultra-pure water was added into a PE disposable paper cup (Figure 2d,h) (simulating the consumption of hot water with a disposable paper cup) and cooled to room temperature naturally. A PET beverage bottle (Figure 2e) was soaked in 4/20 °C ultrapure water for 20 min to simulate the storage of beverage bottles or mineral water bottles at 4 °C or room temperature, since most of these kinds of bottles are made of PET plastic [15,17]. A PPSU baby bottle (Figure 2f) was soaked in 500 mL 50 °C ultrapure water (simulating the process of dissolving milk powder, since generally, it is recommended to use 50 °C hot water for dissolution) for 20 min. PPSU plastic is a common material in the production of baby bottles [18]. Ultrapure water was drawn from an ultrapure water machine with a glass beaker prior to the experiment and stored in glassware throughout the duration of the experiment, thereby minimizing contact with plastic materials. Ultrapure water (with no contact with plastic packaging) at the corresponding temperature was used for the blank control group of the experiment. The dissolution of NPs under different scenarios related to food consumption is shown in Table 1.

All plastic materials used in the experiment were cleaned 5 times with ultrapure water containing detergent, soaked in HCl (pH = 4.0) for 27 h, and cleaned 5 times with ultrapure water to ensure no HCl residue remained, and then all plastic materials were soaked in NaOH (pH = 10.0) and cleaned 5 times with ultrapure water to ensure no NaOH residue remained. Ultrapure water was obtained through an integrated ultrapure water system and stored in non-plastic containers. Finally, processed materials were soaked in ultrapure water for 72 h (water was changed every 6 h and had to be replaced 12 times). The above materials are not easily eroded by conventional acid and alkali. The solution obtained according to the above treatment was filtered step-by-step with polycarbonate membranes with pore sizes of 400, 200, 100, and 50 nm, respectively. The particles retained by the 400 nm membrane were discarded, and NPs ranging from 200 to 400 nm, 100 to 200 nm, and 50 to 100 nm were obtained. A section of 5 × 5 mm^2^ was cut from the center of the filter membrane, and a solid sample was obtained after colloidal silver spraying. A disc with an area of 5 × 5 mm^2^ was cut in the middle of the filter membrane with a clean scalpel and sprayed with metal. Ultrasonic treatment of various polymers has been used to enlarge the size distribution and dispersion of polymer powders in water [19]. The average size of the particles produced through sonication is much closer to the average particle sizes that are relevant in the environment and in weathering studies [20,21]. The remaining part of the filter was put into a beaker with 5 mL ultrapure water and treated with ultrasound for 20 min to obtain a liquid sample. Ultrasound on plastic materials for 20 min might modify the size of NPs and improve dispersibility, which is beneficial for the formation of plastic particles.

### 2.3. Preparation of Colloidal Silver

An amount of 1.67 mM hydroxylamine hydrochloride (HONH_3_Cl) was added to solution with 1 mM AgNO_3_ and 3.33 mM NaOH [22]. The solution was stirred clockwise with a glass rod until it became pale yellow and transparent, and then sealed for storage at 4 °C.

### 2.4. Characterization of NPs

SEM (Gemini Ultra-55; Carl Zeiss AG, Oberkochen, Germany) was employed to observe the surface and section morphology of the NPs (Figure 1). The particle size distribution of sampled NPs was determined using a laser particle size analyzer (ZEN-300, Malvern Panalytical, Malvern, UK). All the ultra-pure water used in the experiment was obtained from an ultra-pure water meter (Pure Force RO-300, Heal force, Hongkong, China). The SERS spectrum of NPs was captured using a confocal Raman microscope (Lab RAM HR Evolution, HORIBA, Irvine, CA, USA), with a He-Ne (633 nm) laser as the source of excitation.

SEM (3 kV) was applied to observe the metal (colloidal silver)-sprayed filter membrane in step 2.2. A 3 mL liquid sample was put in a quartz cuvette for analysis with a particle size analyzer. A 1 mL liquid sample was taken for the SERS test. The operation procedure is as follows: 1 mL liquid sample and 1 mL of the colloidal silver solution prepared in step 2.3 were fully mixed with 50 mL 0.1 mM MgSO_4_ solution. Five μL mixed liquid was added dropwise on a clean silicon wafer, dried at 60 °C for 5 min, and tested by SERS under the conditions of 633 nm and 5% attenuation power.

### 2.5. Data Analysis

Photoshop CS 6.0 software was used to process images. Image Proplus 6.0 software was applied to analyze the SEM images, particle size, and regular degree. Origin 2021 software (v9.8.0.200) was adopted to analyze the data of the particle sizes, Zeta potential, and SERS spectrum.

## 3. Results and Discussion

### 3.1. Morphology Analysis of NPs

**Figure 3:**
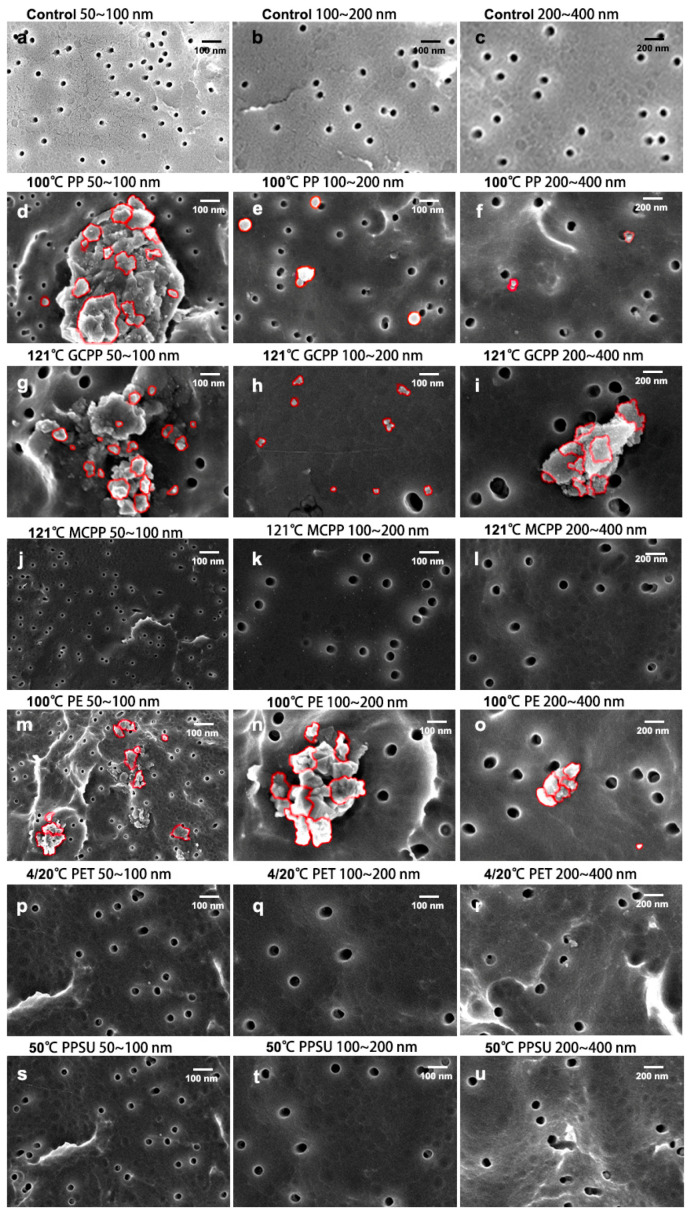
SEM images of the dissolution of NPs in different food consumption scenarios (NPs are marked with red lines). (**a**–**u**) respectively represent the SEM images of samples from each group filtered by three sizes of filter membranes, including the group of ultrapure water at different temperatures, PP plastic soaked in 100 °C hot water, GCPP plastic sterilized at a high temperature of 121 °C, MCPP plastic sterilized at the same temperature, PE plastic soaked in 100 °C hot water, PET plastic soaked in 4/21 °C ultrapure water, and PPSU plastic soaked in 50 °C hot water.

SEM was applied to observe the morphology of the NPs obtained by grading filtration (Figure 3). To avoid the pollution of NPs in ultrapure water, which would interfere with the experimental results, ultrapure water was analyzed by SEM and used as a control sample. Figure 3a–c show similar SEM pictures of ultrapure water at different temperatures after being filtered with different sizes of membranes, and no NPs were observed. Plastics are generally materials with thermal sensitivity, and their robustness can be repeatedly changed by heating. It is reported that elevated temperatures can exacerbate the release of NPs from plastics [23]. The results indicate that ultrapure water was suitable for the control sample. In the PP group treated with 100 °C hot water, NPs were observed in samples filtered through pores of different sizes and became misshapen (Figure 3d–f). In addition, the NPs filtered out with a 50–100 nm filter membrane were obviously aggregated (Figure 3d). Similarly, NPs were observed in the GCPP (Figure 3g–i) and the PE groups (Figure 3m–o) treated with hot water at 121 °C and 100 °C, respectively, and obvious aggregation can be observed in Figure 3g,h,m–o. However, no NPs were observed in the SEM images of the MCPP group (Figure 3j–l), indicating that MCPP plastic food packaging can effectively prevent the dissolution of NPs, although the presence of other toxic substances (such as endocrine disruptors, heavy metals, etc.) cannot be ruled out [24,25,26]. The enhanced effect of temperature on the release of NPs may be due to the thermal or hydrolytic degradation of plastic materials at high temperatures, leading to the subsequent fracture of plastic chains and the cracking and fragmentation of plastic surfaces [25]. Additionally, there were no NPs dissolved in the PET plastic group treated at 4 °C/20 °C (Figure 3p–r), indicating that NPs are not prone to dissolution from mineral water and beverages packaged in PET materials stored in refrigerated conditions or at room temperature. The PPSU group is presented in the process of dissolving milk powder at 50 °C with PPSU milk bottles, and no NPs were observed (Figure 3s–u), which indicates that PPSU is relatively safe to be used as a material for baby bottles.

### 3.2. Measurement Parameters of NP Particles

**Figure 4:**
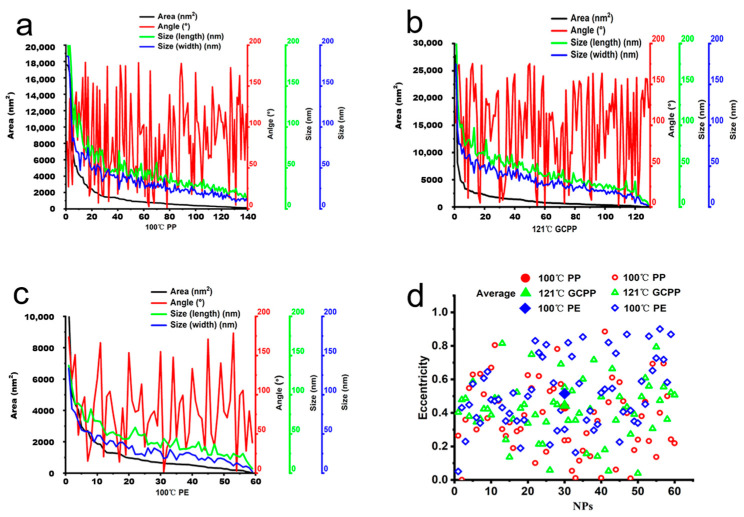
Measurement parameters of the NP particles observed by SEM: (**a**) PP group soaked in 100 °C hot water. (**b**) GCPP group sterilized at high temperature of 121 °C. (**c**) PE group soaked in 100 °C hot water. (**d**) Statistics of eccentricity of NPs. The abscissa represents the number of NPs, and the ordinate represents the eccentricity. The solid symbol indicates the average value of eccentricity, and the hollow symbol indicates the eccentricity of a single NP.

Previous studies have demonstrated that plastic materials release more microplastics at high temperature (60 °C) than that at low temperature (5 °C). This indicates that the release of NPs is dependent on a rise in temperature [27]. Herein, the above SEM results showed that the dissolution of NPs was only found in the groups of PP plastic soaked in 100 °C hot water, GCPP plastic sterilized at a high temperature of 121 °C, and PE plastic soaked in 100 °C hot water. Therefore, further research was carried out only for the above three treatment methods. In the NP measurement parameter graph (Figure 4), the horizontal axis represents the number of NPs, and the black curve refers to the area of the NPs. The red curve represents the angle between the longest and shortest diameters of the NPs, namely, the degree of regularity; the green and blue curves represent the longest and shortest sizes of the NPs, respectively. Based on an analysis of the relevant parameters of the NPs dissolved in the three plastic materials, the parameter values (including area, angle, size, etc.) of all NPs are not uniform, indicating that NPs have the characteristic of irregular shapes (Figure 4a–c). This result is also consistent with previous research reports [28].

Plastic particles have the characteristic of irregularity [29]. To clarify the irregularity of NPs, they are equivalent to an ellipse. Afterwards, we analyzed the eccentricity of the equivalent ellipse. An ellipse with an eccentricity of 0 is considered to be a circle. When the eccentricity is between 0 and 1, this is regarded as an ellipse. As shown in Figure 4d, the eccentricities of the three materials are between 0 and 1, and the eccentricities are 0.435 (100 °C PP), 0.444 (121 °C GCPP), and 0.515 (100 °C PE), illustrating that they are approximately ellipse-shaped. The average eccentricity of GCPP and PE is closer, indicating that their shapes are much more similar.

### 3.3. Particle Size Distribution and Zeta Potential of the NPs

**Figure 5:**
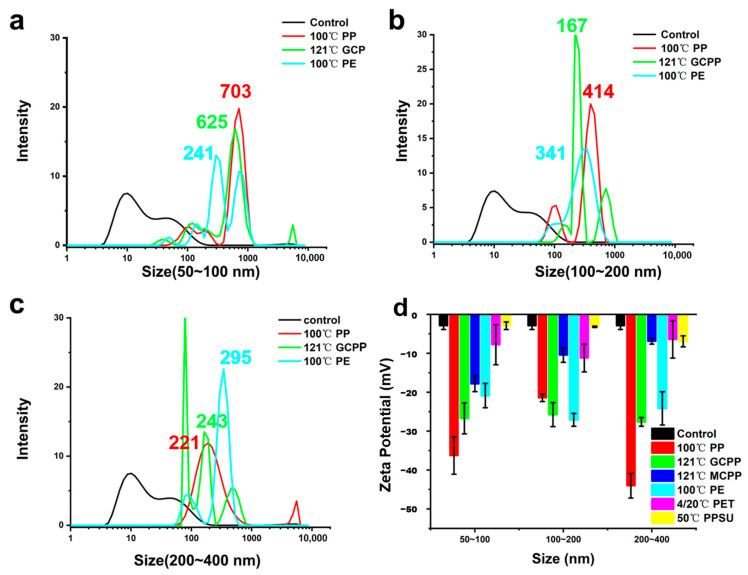
Particle size distribution (**a**–**c**) and Zeta potential (*n* = 3) (**d**) of the NPs filtered by differently sized filter membranes.

In addition, a laser particle size analyzer was applied to measure the particle size distribution of the dissolved NPs. Figure 5 shows the NP particle size distribution of the PP, GCPP, and PE groups filtered by a 50–100 nm (Figure 5a), 100–200 nm (Figure 5b), and 200–400 nm (Figure 5c) filter membrane, respectively. Figure 5a shows that the NPs of the three groups filtered by the 50–100 nm filter membrane are obviously aggregated, and the particle size is 703, 625, and 241 nm, respectively. NPs with a particle size of 50–100 nm will pass through the filter membrane during the filtrating process. Influenced by the unpredictable situation of the accumulation of filtered NPs in water solution, there will be a peak with a large particle size. The particle size of the PE group soaked in 100 °C hot water was distributed at 872 nm (Figure 5a). The particle sizes of the three groups of samples filtered by the 100–200 nm filter membrane were distributed at 414, 168, and 341 nm, respectively, and a certain degree of aggregation appeared (Figure 5b). In addition, part of the particle size in the PE group was also distributed at 488 nm. The particle sizes of the three groups of NPs filtered by the 200–400 nm filter membrane were stabilized at 221, 243, and 295 nm, respectively, and particles around 90 nm and 711 nm appeared in the GCPP group; this is caused by the aggregation of particles. Aggregation is a crucial environmental behavior of NPs, as it determines their mobility and distribution [30]. The aggregation of particles depends on the chance of particle collision and the interaction between colloidal particles [31]. Furthermore, a smaller particle size of NPs will lead to more severe aggregation. Figure 5d shows the result of the Zeta potential. The larger the absolute values of the Zeta potential, the more stable the system. This is consistent with reports showing that a higher absolute Zeta potential value of plastic particles will have less adverse effects on organization (in algae, etc.) [32]. Zeta potential results indicate that the dissolved NPs of the three groups were relatively stable in ultrapure water.

### 3.4. SERS Spectroscopy Analysis of the NPs

**Figure 6:**
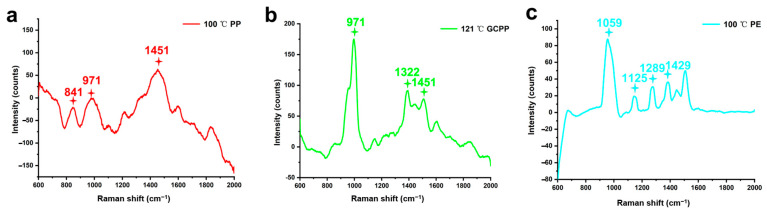
SERS spectra of (**a**) 100 °C PP, (**b**) 121 °C GCPP, (**c**) 100 °C PE group.

The SERS method was adopted to qualitatively detect the dissolved NPs. Dissolved NPs exhibit weak Raman signals. However, dissolved NPs with added colloidal silver generate enhanced Raman signals within the same wavelength range [33]. In ultrapure water, silver nanoparticles prepared with colloidal silver were adsorbed on the surface of the NPs. After being dried, the NPs act as nuclei to attract surrounding silver nanoparticles, and the latter play a role in amplifying signals [34]. After scanning, the characteristic peaks of the PP group were at 841, 971, 1149, and 1451 cm^−1^, that of the GCPP group were at 971, 1322, and 1451 cm^−1^, and in the PE group, they were at 1059, 1125, 1289, and 1429 cm^−1^ (Figure 6a–c). Meanwhile, other measured materials such as MCPP material treated at 121 °C, PET material treated at 4/20 °C, and PPSU material treated at 60 °C were analyzed by SERS, and it was found that the signal was extremely low, so the characteristic peak could not be distinguished. It was verified by SERS spectroscopy that the dissolved NPs of the three groups were made of PP, GCPP, and PE, respectively.

## 4. Conclusions

The consumption of takeaway food is constantly increasing globally, especially in China [35]. Meanwhile, the consumption of hot drinks and heated foods packaged in plastic is gradually increasing among the public, which will promote the release of NPs in the aforementioned food consumption scenarios [27]. This research is focused on the dissolution of NPs in various scenarios related to common food consumption, including eating takeaway food, drinking hot water or beverages, sterilization (high-temperature sterilization) during food production, and dissolving milk powder with high temperatures. SEM images showed that nano-scale particles undergo dissolution upon the immersion of PP or PE plastic in 100 °C hot water, and the GCPP plastic was sterilized at 121 °C. SERS analysis showed that the dissolved nanoscale particles have characteristic peaks of PP, GCPP, and PE, which further confirm that they are NPs. Research has demonstrated that simulated food consumption scenarios do indeed result in the release and production of nanoplastics. Plastic materials treated by high temperature (hot drinks or processed food, etc.) may bring a greater risk of exposure to microplastics than that approached by low temperature. SEM analysis also demonstrated that these NPs are irregular in shape. Meanwhile, it is difficult to fully count the number of NPs during the simulation process, so the specific toxic effects cannot be determined. Therefore, the investigation of the number of NPs released in plastic materials is one of the keys to future study. The presented results show that common plastic consumer products may be a significant source of ingestible nanoparticles that have been identified as a potential risk to the health of biological systems [36]. Therefore, minimizing the use of plastic containers in daily dietary practices can greatly reduce the likelihood of human ingestion of NPs through food consumption. Further investigation into the risks of plastic leakage from food packaging and containers, as well as the exploration of safer materials, would also be invaluable in mitigating health hazards to humans.

## Figures and Tables

**Table 1:** Simulation of representative food consumption scenarios.

Materials	Simulation	Reasons for Selection	Reachable Temperature	Experiment Condition	
				Temperature	Time
PP	Common packaging for hot food with plastic boxes	These six plastic materials are common in food packaging and food consumption scenarios, including industrial food production (GCPP/MCPP), daily foods, beverages (PP/PE/PET), and infant foods (PPSU)	60–100 °C	100 °C	20 min
GCPP	Industrial production prepackaging foods, 121 °C high-temperature sterilization process		100–121 °C	121 °C	20 min
MCPP			100–121 °C	121 °C	20 min
PE	Drinking hot water with embedded PE disposable paper cup, packaging hot food with PE plastic bags		50–100 °C	100 °C	Natural cooling
PET	Common water and beverage plastic bottles		4–20 °C	4/20 °C	20 min
PPSU	Used for dissolving milk powder for infants		50 °C	50 °C	20 min

## Data Availability

Not applicable.
